# The roles of serine protease inhibitors in dermatoses

**DOI:** 10.3389/fgene.2025.1624512

**Published:** 2025-10-03

**Authors:** Zhenzhen Xiao, Yue Kang, Yunqian Zhuo, Rui Li, Yingjian Tan

**Affiliations:** ^1^ Department of Dermatology, Fuzhou First General Hospital, Fuzhou, China; ^2^ Department of Respiratory and Critical Care, Xinxiang Central Hospital, Xinxiang, Henan, China; ^3^ Department of Dermatology, Venereology and Allergology, Charité-Universitätsmedizin Berlin, Berlin, Germany

**Keywords:** serpins, reactive center loop, dermatoses, palmoplantar keratoderma, therapeutic strategies

## Abstract

The balance between proteases and their inhibitors is essential for maintaining the structural and functional homeostasis of the skin. Numerous studies have shown that serine protease inhibitors are highly expressed in the skin and play diverse roles in preserving its physiological integrity. Among them, SERPINs have been closely linked to various skin disorders—for instance, mutations in SERPINB7 are associated with palmoplantar keratoderma, while SERPINA1 has been implicated in the pathogenesis of adult-onset immunodeficiency syndrome and generalized pustular psoriasis, both of which currently have limited treatment options. This review focuses on the biological functions of SERPINs in the skin, aiming to provide insights into the mechanisms underlying SERPIN-related skin diseases and to facilitate the development of targeted therapeutic strategies.

## 1 Introduction

Skin, being the largest organ of the human body, relies on the function of proteases to maintain the stability of its barrier structure and physiological condition (Kishibe, 2019). Positioned on the surface of the body, the skin acts as a natural defense against harmful substances from the external environment (De Veer et al., 2014; Steele et al., 2020). The process of desquamation is intricate, involving the gradual migration of basal keratinocytes towards the stratum corneum under the influence of various regulatory factors. During this process, several proteases hydrolyze structural proteins such as filaggrin, corneodesmosin and desmoglein in the upper epidermis, contributing to desquamation and barrier function. Any abnormalities in the activity of proteases and their inhibitors can disrupt the synthesis and breakdown of these structural proteins, leading to premature or delayed shedding and resulting in parakeratosis or hyperkeratosis (Kishibe, 2019).

SERPINs are the largest protease inhibitor family, with many members and complex functions. They are divided into 16 distinct subfamilies (A-P), with the first 9 classes (A-I) in humans (Gettins, 2002). SERPINs have a highly conserved secondary structure, containing 7-9 α-helices, 3 β-helices, and an exposed reactive center loop (RCL). The tertiary structure of SERPINs is metastable: when the protease cleaves RCL, SERPINs undergo a conformational change from a stressed state to a relaxed state, which is critical to the function of SERPINs (Law et al., 2006). The change in RCL configuration causes a structural change in the active site of SERPINs. Thus, the function of serine protease is inhibited, leading to the loss of the activity of protease hydrolysis. Because the binding of SERPINs and proteases is irreversible, this process is also known as the suicide substrate mechanism (Huntington, 2011). In this case, inhibition relies on the inability of the ends of the scissile bond (P1-P1', according to Schechter and Berger’s nomenclature) to separate after proteolytic cleavage. Consequently, the inhibitor binds tightly, yet reversibly, to a protease without either protein undergoing conformational change. In contrast, serpin RCLs are typically long (20–24 residues) and flexible, resembling a substrate loop (Janciauskiene et al., 2024).

SERPINs are widely expressed in animals, plants, viruses, and other living organisms. So far, more than 1,500 species have been found, 37 of which are found in the human body. A host of SERPIN genes are clustered on the same chromosome, especially chromosomes 14 and 18 (Aslam and Yuan, 2020). Within each cluster, all SERPINs genes belong to the same clade. For instance, the clusters of genes on chromosomes 18 entirely belong to clade B, which indicates these genes evolved from a common ancestor by chromosomal duplications. Interestingly, despite their chromosomal proximity, the functions of these genes vary greatly. SERPINs can be secreted into the cytoplasm, nucleus, and even the extracellular environment and exert a protease inhibition effect in the main suicide substrate mechanism in the human body. However, some SERPINs do not act as protease inhibitors but participate in inflammation, anticoagulation, antitumor, and other processes. Of note, numerous skin diseases have been attributed to SERPINs monogenetic mutations, most of which are deleterious.

Emerging evidence has highlighted the involvement of SERPINs in the regulation of key intracellular signaling pathways that orchestrate inflammatory and stress responses in the skin. For instance, SERPINB3 and SERPINB4 have been shown to suppress the activation of the NF-κB and MAPK pathways in keratinocytes, thereby limiting the expression of pro-inflammatory cytokines such as IL-6 and IL-8 (Sun et al., 2017). Additionally, SERPINA1 (α1-antitrypsin) has been reported to inhibit STAT3 phosphorylation in models of cutaneous inflammation, suggesting broader immune-modulatory effects (Kantaputra P. et al., 2021). *In vivo* studies further substantiate these findings. Transgenic mice overexpressing SERPINB3 exhibit reduced epidermal inflammation and improved barrier recovery following irritant exposure, whereas SERPINB3 knockout models demonstrate exacerbated skin inflammation and increased protease activity (Sivaprasad et al., 2015). Likewise, SERPINE1-deficient mice show delayed wound healing and enhanced matrix metalloproteinases (MMPs) activation, reinforcing its role in protease balance and tissue remodeling (Chen et al., 2021). These *in vivo* models provide mechanistic insight into how SERPINs maintain skin integrity and modulate inflammatory responses under physiological and pathological conditions.

A recent integrative study combining tissue proteomics and the public cutaneous squamous cell carcinoma(cSCC) transcriptomic dataset GSE32628 identified 20 overlapping genes and validated eight proteins (TNC, FSCN1, SERPINB1, ACTN1, RAB31, COL3A1, COL1A1, CD36) as differentially expressed between Bowen’s disease, cSCC and normal skin (Biao et al., 2022); importantly, SERPINB1 was functionally interrogated by siRNA in A431 cells, where its knockdown reduced migration and invasion, providing direct proteomic-to-functional evidence linking a SERPIN to invasive behaviour. Independent transcriptomic and proteomic literature further supports involvement of the SERPIN family across cutaneous disorders: elevated SERPINB3/B4 expression has been reproducibly observed in inflammatory skin diseases and implicated in barrier dysfunction and early inflammation, consistent with transcript-level dysregulation observed in RNA-seq studies (Sivaprasad et al., 2015). Genetic and transcriptomic analyses in neutrophil-rich pustular dermatoses have also identified SERPINA1 and SERPINA3 as disease-relevant (including loss-of-function variants that perturb protease inhibition and IL-36 regulation), illustrating that both sequence variation and expression changes of SERPINs can contribute to cutaneous inflammatory pathology (Yang et al., 2023). More broadly, proteomic profiling of keratinocytic lesions and recent multi-omics reviews demonstrate that proteome and transcriptome datasets can discriminate lesion types (actinic keratosis, Bowen’s disease, cSCC) and uncover pathways (ECM–receptor interaction, focal adhesion, PI3K-Akt signaling) in which SERPINs participate, thereby providing systems-level context for the specific SERPIN alterations highlighted above (Rusiñol and Puig, 2024). Taken together, these transcriptomic, proteomic and genetic studies—centered on the Tang et al. proteomics–GEO integration and supported by independent omics and genetic reports—supply the high-throughput evidence the reviewers requested and justify the inclusion of SERPINB1, SERPINB3/4, SERPINA1/3 and related proteins in our review as candidate mediators and biomarkers of progression from Bowen’s disease to invasive cSCC (Biao et al., 2022).

An intricate regulatory relationship exists between serine protease inhibitors (SERPINs) and MMPs in maintaining skin homeostasis and responding to pathological stimuli. MMPs, including MMP-1, MMP-2, and MMP-9, are essential for extracellular matrix (ECM) remodeling, keratinocyte migration, and inflammation resolution. Dysregulated MMP activity has been implicated in various skin disorders, such as chronic wounds, psoriasis, and atopic dermatitis, often resulting in excessive ECM degradation and barrier dysfunction (Ohtomo et al., 2008). SERPINs, traditionally recognized for their roles in inhibiting serine proteases, have emerged as indirect regulators of MMPs. For example, SERPINE1 can modulate MMP activation by limiting plasmin generation, which is required for the proteolytic activation of latent MMPs (Chen et al., 2021). Moreover, studies suggest that SERPINB3 and SERPINB4 may influence MMP expression through modulation of oxidative stress and inflammatory pathways (Sun et al., 2017). The loss of balance between SERPIN-mediated inhibition and MMP-driven proteolysis may contribute to exaggerated inflammation, impaired barrier repair, and tissue remodeling in inflammatory skin diseases. Further elucidation of the SERPIN–MMP axis may offer novel therapeutic opportunities for modulating protease activity in skin pathology.

In this study, we reviewed (Table 1) the clinical manifestation and pathogenesis of skin diseases related to SERPINs in order to improve our understanding of the potential role of SERPINs (Maas and de Maat, 2021). Due to numerous biological functions and pathological states associated with SERPINs, further characterization of the structure and mechanism information of SERPINs will shed light on the therapeutic targets of SERPIN-related skin diseases.

**TABLE 1:** Expression and target proteases of human serpins related to skin diseases.

SERPIN name	Chromosomal location	Aliases	Target protease	Expression pattern	Main functions in skin
SERPINA1	14q32.13	Antitrypsin	Inhibition of neutrophil elastase (Chua et al., 2009)	Liver, bone marrow, the lymphocytic and monocytic cells in lymphoid tissue, the Paneth cells of the gut, neutrophils	Loss of function mutations in SerpinA1 activates IL-36a, contributing to the development of GPP (Chua et al., 2009)
SERPINA3	14q32.1	Antichymotrypsin(ACT)	Inhibition of chymotrypsin, cathepsin G	Liver, gall bladder, brain, prostate, testis, pancreas	Loss of function mutations in SerpinA3 activates IL-36 g, contributing to the development of GPP (Martinoli et al., 2014)
SERPINA12	14q32.13	Vaspin	Inhibition of kallikrein 7 (KLK7) and 14 (KLK14) (Yang et al., 2023; Rusiñol and Puig, 2024)	Keratinocyte (Yang et al., 2023)	SERPINA12 mutants lead to the palmoplantar keratoderma (Ohtomo et al., 2008). SERPINA12 inhibits the inflammation in psoriasis (Biao et al., 2022)
SERPINB3	18q21.3	SCCA1	Inhibition of cathepsin L, S, K and papain (Blaydon et al., 2011)	Esophagus, urinary bladder, skin, immune cells and many mucosal cells	Both of them express increasely in squamous cell carcinoma (SCC), atopic dermatitis (AD), and psoriasis lesions, and play a pro-inflammatory role (Haslund et al., 2019)
SERPINB4	18q21.3	SCCA2	Inhibition of chymase, Cathepsin G, Granzyme M (Blaydon et al., 2011)	Esophagus, urinary bladder, skin, appendix	
SERPINB5	18q21.3	PI5; maspin	Non	Esophagus, skin, urinary bladder, small intestine	Decrease in SERPINB5 is associated with regional lymph node metastasis of melanoma (Levi et al., 2019)
SERPINB6	6p25	PI6	Inhibition of cathepsin G, plasmin, thrombin and kallikrein- 8) (Ribero et al., 2017)	Blood cells, platelets, endothelial cells, keratinocytes and other epithelial cells, widely expressed in human tissues	SERPINB6 is associated with wound healing, cell migration, cell proliferation (Ribero et al., 2017)
SERPINB7	18q21.33	Megsin	Inhibition of LGMN (Hannula-Jouppi et al., 2020)	Mesangial cells, skin (Saalbach et al., 2012; Hannula-Jouppi et al., 2020)	SerpinB7 deficiency can cause epidermal barrier dysfunction in psoriasis (Shimomura, 2024). SERPINB7 mutants lead to the Nagashima-type palmoplantar keratoderma by increasing the enzyme activity of LGMN (Hannula-Jouppi et al., 2020)
SERPINB8	18q21.3	PI8	Inhibition of furin	Epithelial layer of skin appendages; Nuclei of squamous epithelium of mouth, pharynx, esophagus, and epidermis; monocytes and neuroendocrine cells in the pituitary gland, pancreas, and digestive tract	SERPINB8 mutants can cause the exfoliative ichthyosis (Liu et al., 2023)
SERPINB13	18q21.33	Hurpin	Inhibition of cathepsin L and K (Katagiri et al., 2006)	Esophagus, skin, urinary bladder, blood, kidneys and saliva	SERPINB13 can interfere with UVB-induced keratinocyte apoptosis by interacting with cathepsin L (Katagiri et al., 2006)
SERPINE1	7q22.1	Plasminogen activator inhibitor-1(PAI1)	Inhibition of thrombin, tissue plasminogen activator (tPA), urokinase (uPA) (Blaydon et al., 2011)	Gall bladder, placenta, liver	SERPINE1 is involved in the PLAUR-TLR2-OSM pathway, which induces AD-like skin lesions in mice and promotes chronic pruritus (Blaydon et al., 2011)
SERPING1	11q12.1	complement I esterase inhibitor; C1 inhibitor(C1IN)	Inhibition of Complement I esterase (Ohguchi et al., 2018)	Liver, gall bladder, ovary, skin	Loss of function mutations in SERPING1 can cause the HAE (Pasmooij, 2018)

## 2 SERPINs related to keratosis

**FIGURE 1:**
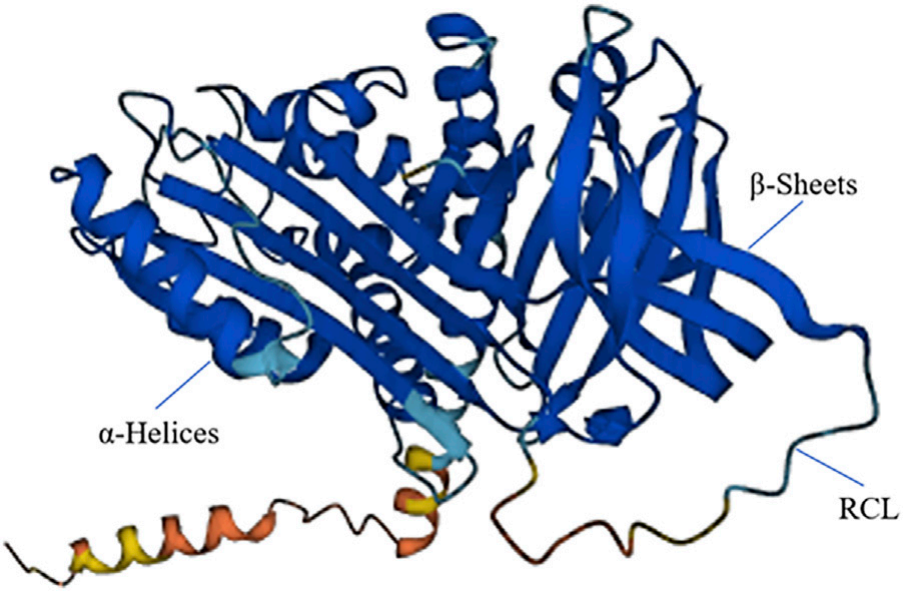
The 3D structure of SERPINA12. SERPINA12 with labeled structural elements: α-helices, β-sheet and reactive center loop (RCL).

*SERPINA12* encodesthe VASPIN (Visceral Adipose Tissue-Derived Serine Protease Inhibitor) protein, which consists of 414 amino acids. VASPIN comprises 3 beta folds, 9 alpha helices, and a RCL structure located between amino acids G364 and P381 at the C-terminal (Figure 1). The RCL region is identified and cleaved between the M378 (P) and E379 (P1′) sites to form a stable complex (Weiner et al., 2019; Kurowska et al., 2021). Although VASPIN was initially discovered in human fat tissue and its association with diabetes, its presence in skin was first reported in 2012 (Hida et al., 2005). VASPIN is predominantly expressed in the suprabasal layerof the epidermis. Studies by Saalbach et al. (2012) revealed lower VASPIN expression in psoriatic skin lesions compared to non-psoriatic skin lesions. However, VASPIN expression was found to be higher in differentiated cells within psoriatic skin lesions. When HaCaT cells were co-cultured with immune cells, VASPIN treatment demonstrated the ability to modulate inflammatory phenotypes in psoriasis models. These findings suggest that VASPIN may play a role in regulating inflammation during the development of psoriasis, making it a potential target for psoriasis treatment. VASPINfunctions as an inhibitor of serine proteases, but it does not inhibit common serine proteases such as pancreatic enzymes. It is known to inhibit kallikrein 7 (KLK7) and KLK14 (Heiker et al., 2013; Ulbricht et al., 2018). In 2020, Mohamad et al. (2020) originally reported two cases of autosomal recessive palmoplantar keratoderma (PPK) patients with *SERPINA12* mutations, including c.631C>T(p.Arg211Ter) and c.1051G>T(p.Glu351Ter). Both of the variants are homozygous nonsense variants, leading to the lower expression and the loss of its function. Mohamad et al found that VASPINregulates the process of skin desquamation by inhibiting the protease activity of KLK7. KLK7 activity is markedly increased after the loss of *SERPINA12* mutation and increases the proteolytic degradation of DSG1 and CDSN, which is necessary for normal cornification and epidermal differentiation (Steele et al., 2020; Mohamad et al., 2020). Subsequently, additional studies have described cases of SERPINA12-related PPK. In 2022, Liu et al. (2022) reported six autosomal recessive PPK patients caused by *SERPINA12* mutations and identified four new mutations: c.970_971del, c.662delA, c.656A>G, and c.635-7A>G. In population genetics, a founder effect refers to the reduced genetic diversity that results when a population is descended from a small number of ancestors. This can lead to the increased frequency of rare genetic variants, including pathogenic mutations, within specific populations. Haplotype analysis results of the c.970_971del mutation, which had the highest frequency among these mutations, suggest that *SERPINA12* c.970_971del may be a founder mutation. Patients carrying this mutation share a common haplotype with a distance of at least 17kb. Recently, Hannula-Jouppi (Brandt et al., 2024) lab identified a new *SERPINA12* c.1100G>A (p.Gly367Glu) missense variant. And a previously reported *SERPINA12* c.631C>T (p.Arg211*) variant is carried by all three patients, and they share a 53 kilobases haplotype, which suggesting c.631C>T may be a founder mutation. Collectively, seven distinct *SERPINA12* variants have been reported as the cause of PPK, including c.970_971del, c.662delA, c.656A>G, c.635-7A>G, c.631C>T, c.1051G>T, c.1100G>A. Interestingly, the *SERPINA12*-related PPK patients reported by Hannula-Jouppi et al. (2020) all carry a heterozygous *SERPINB7* c.1136G>A (p.Cys379Tyr) variant, which suggests the potential connection between *SERPINA12* and *SERPINB7*.

**FIGURE 2:**
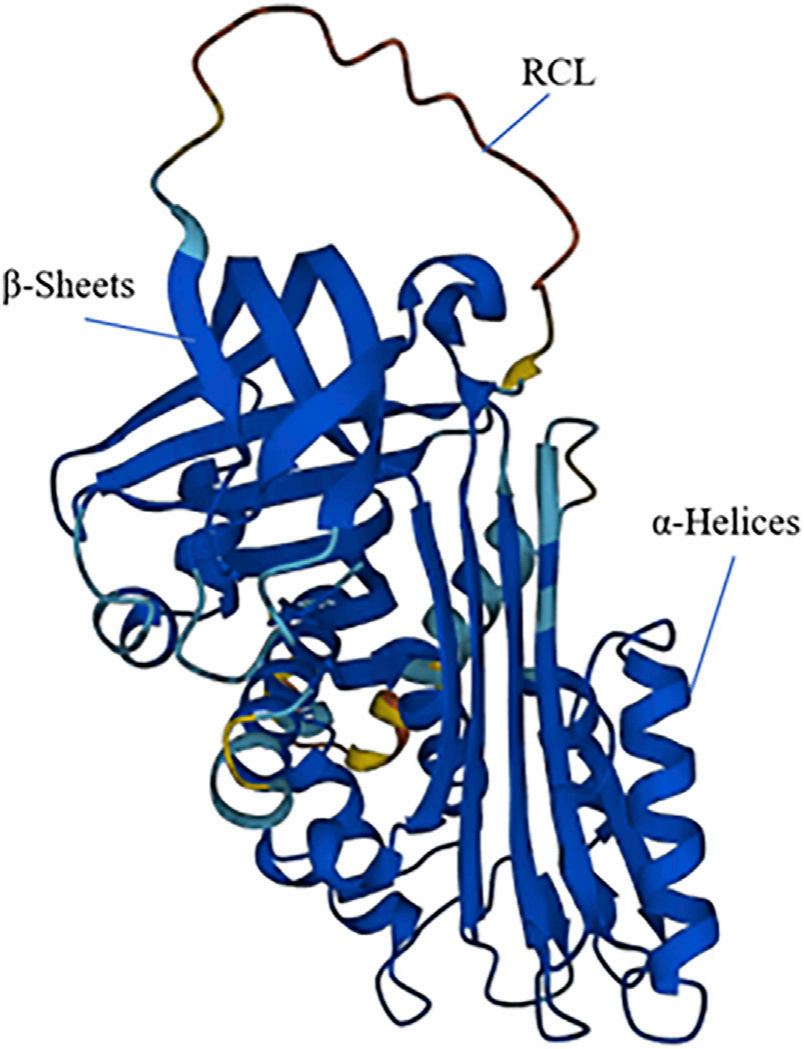
The 3D structure of SERPINB7. SERPINB7 with labeled structural elements: α-helices, β-sheet and reactive center loop (RCL).

*SERPINB7* encodes the MEGSIN protein, which consists of 380 amino acids (Figure 2). In 1998, Kurokawa (Miyata et al., 1998) first cloned the *SERPINB7* gene from human mesangial cells. By comparing its amino acid sequence with other proteins, it was determined that *SERPINB7* belongs to the serine protease inhibitor family and exhibits the characteristic structural features of the *SERPIN* family, including the RCL structure. MEGSIN is primarily expressed in human mesangial cells, and its expression is notably elevated in the mesangial cells of patients with IgA nephropathy. In 2002, Miyata et al. (2002) created a mouse model that overexpressed Serpinb7. This led to distinct changes in the mesangial matrix within the kidneys of the mice. There was an expansion in the matrix and an increase in the number of mesangial cells, and *in vitro* experiments confirmed that Megsin could inhibit plasminase.

The inhibitory function of MEGSINon proteases suggests its potential role in regulating fibrinolysis, a process involved in the pathogenesis of IgA nephropathy. In 2008, Toshio Miyata et al. (Ohtomo et al., 2008) discovered that MEGSIN can reduce the protease activity of MMP-1 and MMP-9. MMP-1 and MMP-9 are metalloproteinases that can degrade essential components of the mesangial extracellular matrix, including type IV collagen, laminin, and fibronectin, which play a role in regulating the synthesis and degradation of the extracellular matrix. MMP-1 and MMP-9 exist in an inactive proenzyme state and require activation by plasminase. MEGSINinhibits plasminase, and the inability of MMP-9 to activate may contribute to the mechanisms by which MEGSINis involved in diabetic nephropathy. In 2013, Kubo et al. (2013) identified *SERPINB7* as the causing gene of autosomal recessive Nagashima-type Palmoplantar Keratoderma (NPPK) through whole exon sequencing of samples from three unrelated Japanese patients, and confirming *SERPINB7* c.796C>T as a founder mutation through haplotype analysis. NPPK is characterized by the presence of clear erythema in the palmar and plantar areas, often accompanied by mild diffuse hyperkeratosis that may extend to the backs of the hands, feet, and the inside of the wrists. NPPK is more prevalent in east Asia and is the most common type of palmoplantar keratoderma in the Chinese Han population (Shimomura, 2024). Hannula-Jouppi et al. (2020) indicates that NPPK, an autosomal recessive diffuse PPK caused by *SERPINB7* variants resulting in excessive protease activity, exhibits resemblances to SERPINA12-related PPK. *SERPINB7* may lead to PPK through the similar mechanism of PPK caused by the *SERPINA12* mutation. A group of total 234 Chinese NPPK patients were established, which would enhance the current understanding of NPPK, expand the mutation spectrum of *SERPINB7,* and offer evidence-based recommendations for future clinical management, diagnosis, and genetic counseling of NPPK patients (Liu et al., 2023). At present, seventeen different *SERPINB7* variants has been identified, including c.122_127del, c.218_219delins, c.271delC, c.336+2T>G, c.382C>T, c.434G>C, c.455-1G>A, c.455G>A, c.455G>T, c.522dup, c.530T>C, c.635del, c.643A>G, c.650_653del, c.796C>T, c.830C>T, c.1136G>T (Brandt et al., 2024). Among these mutations, c.796C>T is the most frequent and proposed to be enriched in east Asian populations for founder effect. Besides, c.218_219delins and c.830C>T are relatively more common than other variants. Recent study (Liu et al., 2024) describes the potential digenic inheritance of *SERPINB7* and *SERPINA12* variants, shed light on the relationship between these two important serine protease inhibitors.

One recent study (Li et al., 2024) showed that the mutation of *SERPINB7* is the cause of NPPK through inhibiting the protease legumain. MEGSINbound directly with legumain and inhibited legumain activity in a ‘protease-substrate' manner at the cleavage sites of MEGSINas Asn^71^ and Asn^343^. This research demonstrated that MEGSINdeficiency results in the overactivation of legumain, which may further compromise the epidermal barrier, leading to NPPK phenotypes. These findings suggest that legumain could be a promising therapeutic target for NPPK. Recent studies have contributed to a better understanding of the regulation of protease and inhibitor networks in the skin, shedding light on their potential roles in the pathogenesis of keratoderma.

Current treatments for this condition typically involve the topical use of moisturizer for symptom relief. Ohguchi et al. (2018) conducted clinical trials and confirmed that the administration of gentamicin can induce ribosome read-through of the *SERPINB7* c.796C>T nonsense mutation. Gentamicin causes the ribosome to read through the stop codon and the production of full-length MEGSINprotein, leading to a significant improvement in patients’ phenotypes. The mechanism involves nonsense mutation-mediated mRNA decay (NMD) and 26S proteasome-mediated protein degradation, preventing the production of truncated proteins (Pasmooij, 2018; Wang et al., 2023).

*SERPINB7* has also been reported to function as a key signaling molecule during the pathogenesis of psoriasis (Zheng et al., 2022), which is abundantly expressed in the lesions of psoriasis patients and imiquimod-induced psoriatic mouse model. *SERPINB7* deficiency inhibited keratinocyte differentiation and increased inflammation by diminishing the calcium concentration in the cytosol and mitochondria, contributing to the development of psoriasis. The deficiency of *SerpinB7* impairs skin barrier function and modulates the expression of inflammatory mediators in mice, thereby exacerbating psoriasis-like lesions. Transcriptomic and proteomic analyses reveal that the absence of *SerpinB7* disrupts keratinocyte differentiation and alters the expression of genes associated with the calcium signaling pathway. Experimental data confirm that the loss of *SerpinB7* reduces calcium ion concentration in keratinocytes, leading to inhibited differentiation of these cells. This inhibition subsequently promotes the expression of keratinocytes and inflammatory mediators, thereby influencing the onset and progression of psoriasis.

*SERPINB8* encodes a protein consisting of 374 amino acids. In 2016, Pigors et al. (2016) reported a series of cases of exfoliative ichthyosis in three unrelated families caused by loss-of-function mutations in *SERPINB8*. These mutations included *SERPINB8* c.947delA (p.Lys316Serfs90), c.850C>T (p.Arg284), and c.2T>C (p.Met1?). Cell experiments confirmed an upregulation in the expression of desmoplakin isoforms I and II as well as desmoglein-1 proteins in HaCaT cells after *SERPINB8* knockdown. The specific molecular mechanisms through which *SERPINB8* mutations lead to exfoliative ichthyosis remain to be explored. It's worth noting that *CSTA* mutation of c.67-2A>T and c.256C>T can also lead to exfoliative ichthyosis (Blaydon et al., 2011), indicating a correlation between *SERPINB8* and *CSTA*, which could involve an upstream or downstream relationship, or they may share the same enzyme substrates, potentially contributing to a similar disease.

## 3 Hereditary angioedema caused by *SERPING1* mutation

*SERPING1* encodes the complement 1 esterase inhibitor (C1 esterase inhibitor, C1-INH) protein, which plays a crucial role in the regulation of complement activity. Classic hereditary angioedema (HAE) is primarily caused by mutations in *SERPING1*, making it the most common type of HAE (Haslund et al., 2019). As previously reviewed, more than 700 distinct *SERPING1* variants have been reported to result from HAE. Among these mutations (Sinnathamby et al., 2023; Santacroce et al., 2021), a host of genotypes are observed, including missense variants (32.1%) and nonsense variants (9.0%), short deletions, duplications, and delins variants (36.2%), splice defects (13.6%), large deletions and duplications (8.2%), and a few variants affecting 5′‐untranscribed and 3′‐untranslated region (3′‐UTR) sequences (0.9%). HAE is characterized by acute, recurrent subcutaneous and submucosal edema. Clinical manifestations can affect various parts of the body, including the face, limbs, trunk, digestive tract, and respiratory tract, and can be life-threatening in severe cases. *SERPING1* mutations can lead to two main types of HAE: type I HAE, accounting for approximately 85% of classic HAE cases, results from reduced expression of C1-INH, leading to insufficient function, and type II HAE, accounting for about 15% of classic HAE cases, is characterized by normal expression but lacking function of C1-INH (Betschel et al., 2023). Complement C1 is a vital serum protein involved in regulating phagocyte function and mediating inflammatory responses, including increasing vascular permeability. *SERPING1* mutations decrease the inhibitory function of C1-INH, leading to overactivation of C1. Activated C1 can cleave downstream substrates, including complement C2 and C4, resulting in reduced blood vessel concentrations of C2 and C4 (Levi et al., 2019).

Loss of C1-INH function also affects coagulation factor XII, which activates clotting factor XIIa, promoting the conversion of prokallikrenase into kallikrenase. Through positive feedback, this leads to increased levels of clotting factor XIIa. Kallikrein binds to the bradykinin β2 receptor, leading to vasodilation and increased blood vessel permeability, which can result in skin and mucosal edema. In addition to *SERPING1* mutations, HAE can also be caused by mutations in the *F12*, *PLG*, and *ANGPT1* genes (Levi et al., 2019). However, the precise pathogenesis of HAE is still not fully understood. Understanding the genetic basis of HAE, including the role of genes such as *SERPING1*, is crucial for accurate diagnosis and subsequent treatment of this condition. HAE is a life-threatening disease required immediate and effective therapies especially when it involves the upper airway.

HAE is primarily driven by the pro-inflammatory mediator bradykinin and results from a deficiency of the liver-produced acute-phase reactant C1-inhibitor protein. The C1-inhibitor protein limits the activation of C1r, C1s, MASP-1, and MASP-2, thereby inhibiting the complement system. When C1-inhibitor protein is nonfunctional or present in low levels, unrestrained complement activity occurs, leading to increased vascular permeability and edema (Sinnathamby et al., 2023). Furthermore, the C1-inhibitor protein also inhibits plasma kallikrein, the enzyme responsible for converting high-molecular-weight kininogen (HMWK) to bradykinin. Low levels of C1-inhibitor allow plasma kallikrein levels to rise, subsequently increasing bradykinin levels. Notably, plasma kallikrein enhances the activation of factor XII to factor XIIa, which further promotes the conversion of prekallikrein to kallikrein, thus boosting bradykinin production. Bradykinin is responsible for many HAE symptoms, as it induces endothelial contraction, nociceptor activation, and bronchoconstriction, leading to edema, pain, and dry cough typical in HAE patients. It is worth noting that angiotensin-converting enzyme (ACE) reduces bradykinin levels. Therefore, ACE inhibitors, which are effective anti-hypertensive agents, can induce HAE, a condition known as ACE-induced HAE (Sinnathamby et al., 2023; Busse and Christiansen, 2020).

## 4 SERPINs associated with skin tumors

Squamous cell carcinoma antigen 1 (*SCCA1*) and squamous cell carcinoma antigen 2 (*SCCA2*) were encoded by *SERPINB3* and *SERPINB4*, respectively. The base sequences of *SERPINB3*and *SERPINB4*are highly homologous. However, the RCL sequences of the two genes are distinct, and the inhibitory substrates are also different. SERPINB3 mainly inhibits cathepsin K, L, S, and V, while SERPINB4 primarily inhibits CTSG, Granzyme M and chymase (Sun et al., 2017). Studies have shown that the expression of SERPINB3 and SERPINB4 increases in SCC, atopic dermatitis, and psoriasis lesions, and they can play a pro-inflammatory role (Ren et al., 2020). Research conducted by Katagiri et al. revealed that the expression of SERPINB3/4 increased in UV-irradiated epidermis. This increase inhibited the phosphatase activity of JNK1 and ultimately suppressed UV-induced autophagy (Katagiri et al., 2006). Sivaprasad et al. (2015) observed an elevation in SERPINB3/4 expression in the skin lesions of patients with AD. Furthermore, the knockout of SERPINB3/4 inhibited the acute inflammatory response in mouse models of AD, indicating their proinflammatory effect in chronic inflammatory skin diseases.

*SERPINB5* encodes the MASPIN protein which consists of 375 amino acids. It functions as a regulator to suppress the tumor metastasis, such as melanoma, without serine protease activity inhibitory effect (Chua et al., 2009). Melanoma is a malignant skin tumor characterized by frequent local recurrence and early metastasis. A study of mass spectrometry-based proteomics on a variety of melanoma samples shows that the decrease in *SERPINB5* is associated with regional lymph node metastasis of melanoma (Martinoli et al., 2014). In primary melanomas, nuclear MASPIN expression was correlated with disease stage and melanoma prognostic factors (tumor thickness, mitotic rate, nodular histotype, and ulceration). In contrast, cytoplasmic MASPIN was more frequently seen in thin superficial spreading melanomas that did not undergo mitosis (Ribero et al., 2017).

## 5 SERPINs associated with common inflammatory skin diseases

SERPINs play a role in mediating the release of inflammatory factors during the development of common inflammatory skin diseases, such as AD and psoriasis. Besides the *SERPINA12*, *SERPINB7*, *SERPINB3*, *SERPINB4*, et al., other SERPINs are also correlated with the pathogenesis of AD and psoriasis (Morizane et al., 2022). *SERINE11* and *SERPINB13* are involved in the pathogenesis of inflammatory skin diseases while no diseases caused by the monogenic mutation of these two genes have been determined.

*SERPINE1* encodes a plasminogen activator inhibitor type-1(PAI-1) comprising 402 amino acids. *SERPINE1* is dermally overexpressed in the lesions of AD patients, and TRPV3 activation induces the release of SERPINE1 from the primary keratinocyte (Sun et al., 2017). As previously reported, intradermal injection of SerpinE1 induced an itch-like behavior in AD mouse model, and its antagonist ameliorated itch, implicating its role in AD patients with chronic itch. In addition, *SERPINE1* is involved in the PLAUR-TLR2-OSM pathway, which induces AD-like skin lesions in mice and promotes chronic pruritus. PAI-1 promotes fibrosis through the lytic activation of tissue plasminogen activator (t-PA) and urokinase plasminogen activator (u-PA). Elevated expression of *SERPINE1* temporally and spatially plays a role in proteolytic activity, facilitating the invasive potential of squamous cell carcinoma, and is involved in wound healing, cell migration, cell proliferation, and other processes. PAI-1 is increasingly expressed in the skin, especially the epidermis and microvessel endothelium, in patients with systemic sclerosis. PAI-1 neutralization can induce plasmin-mediated metalloproteinase 1 activation and then reduce the initial vascular injury and subsequent tissue inflammation, leading to the improvement of skin fibrosis.

In addition, PAI-1 upregulates the intercellular adhesion molecule type 1 in skin fibroblasts and binds to mast cells. The fibroblast–mast cell interactions result in the activation of the adherent mast cells, which secret cytokines compromising IL-4 and IL-13, contributing to fibrogenesis. These studies highlight the potential role of PAI-1 in fibrosis-related diseases such as systemic sclerosis, wound healing, et al.

*SERPINB13* encodes the HURPIN protein, which comprises 391 amino acids. In normal human skin, *SERPINB13* is expressed in the basal layer. However, in disease lesions like AD, psoriasis, and squamous cell carcinoma, its increased expression is observed in the spinous and granular layers. HURPINspecifically inhibited cathepsins K and L with a serpin-like mechanism. Through differential gene expression analysis, researchers have observed the downregulation of *SERPINB13* after UVB irradiation of HaCaT cells and *SERPINB13* can interfere with UVB-induced keratinocyte apoptosis by interacting with cathepsinL. Deletion of *SERPINB13* serves to induce the overactivation of CTSL (Welss et al., 2003), which generates caspase 3 and thus contributes to apoptosis. In addition, *SERPINB3* is also designated as the differentiation marker in psoriasis, squamous cell carcinoma, and other skin diseases.

## 6 The role of SERPINs in other skin diseases

Generalized pustular psoriasis(GPP) is a pustular autoinflammatory skin disease characterized by acute generalized erythema and scaling with numerous aseptic pustules. It shares a similar skin manifestation with the autoimmune disease adult-onset immunodeficiency (AOID), which is induced by the overproduction and overresponse of IFN-γ. AOID is characterized by patients always having high titers of anti-IFN-γ autoantibodies, strongly correlated with higher severity.

Numerous gene mutations have been identified to cause the AOID and GPP, including *IL36RN*, *CARD14*, *IL1RN*, *AP1S3*, *MPO*, *TNIP1*, and *SERPINs*. Variants in SERPIN genes resulting in AOID and GPP indicate that the function of SERPINs is involved in the pathogenesis of these diseases, and genetic investigation is necessary for further diagnosis and treatment of these two diseases.

*SERPINA1* encodes a protein comprising 418 amino acids. Two unrelated patients were reported, one with adult-onset immunodeficiency syndrome (AOID) and a pustular skin reaction and the other with GPP, both of whom carried a heterozygous missense *SERPINA1* mutation, c.718G>A (p.Val240Met) (Kantaputra P. et al., 2021). *SERPINA1*, one significant member of the serine protease family, plays a vital role in proteolytic activity. The *SERPINA1* mutation leads to reduced inhibition of elastase and cathepsin G, resulting in subsequent amplification of inflammatory reactions with neutrophil recruitment. Besides inhibiting protease activity, *SERPINA1* can also regulate neutrophil-driven autoimmunity. Deficiency of *SERPINA1* function has been determined to increase the level of neutrophilic TNF-α, resulting in neutrophil degranulation. Collectively, these mechanisms could explain the pathogenesis of the *SERPINA1* mutation leading to AOIDand GPP, respectively. *SERPINA1* shares almost 40%–45% of its amino acids and inhibitory functions with *SERPINA3*, which is also associated with AOIDand GPP.

*SERPINA3* encodes alpha-1-antichymotrypsin (ACT), comprising 423 amino acids. The RCL region of SERPINA3 encompasses amino acid sites 378–395. *SERPINA3* inhibits cathepsin G and chymotrypsin. *SERPINA3* c.966delT(p.Tyr322Ter) was determined to be the cause of GPP patients through nonsense-mediated mRNA decay and therefore haploinsufficiency of *SERPINA3* (Kantaputra P. N. et al., 2021). The loss of *SERPINA3* inhibitory function causes the overactivation of neutrophilic serine proteases, especially cathepsin G. In addition, four rare variants of *SERPINA3* resulting in skin pustules and adult-onset immunodeficiency have been reported, including c.684G>C, c.923T>C, c.1240A>G, and c.1246_1247del. The first three are missense mutations, and the last one is a deletion mutation. The inhibitory effect of *SERPINA3* with loss-of-function variants on cathepsin G decreased, contributing to the GPP.

In 2023, Kantaputra P. N. et al. (2021) reported that the heterozygous missense variant *SERPINA1* c.438G>T (p.Lys146Asn) was determined in two patients with AOID and GPP, respectively. In addition, *SERPINA3* c.917A>G (p.Asp306Gly) is also reported with AOID (Kantaputra P. et al., 2021). Immunohistochemistry shows increased expression of *SERPINA1* and *SERPINA3* in the skin of patients, compared to a lower level in normal people. Due to the same causing gene of *SERPINA3*, GPP and AOID appear to share pathogenetic mechanisms. The main substrate of *SERPINA3* is cathepsin L, which can inactivate *SERPINA1* and lead to the subsequent inflammatory response and autoimmune reaction.

*SERPINB6* encodes a protein containing 376 amino acids, which is namedplasminogen activator inhibitor 2 (PAI-2). Plasminogen activator inhibitor type-2 (PAI-2) co-activates tissue plasminogen activator (t-PA) and urokinase plasminogen activator (u-PA). It also plays a role in the pathogenesis of wound healing, cell migration, cell proliferation, and other processes. In 2007, Scott et al. (2007) discovered that *SERPINB6* is co-expressed with KLK8 in the basal layer of human skin. Co-immunoprecipitation experiments confirmed that SERPINB6 can bind to KLK8, forming a complex that inhibits KLK8 protease activity. In 2010, Sirmaci et al. (2010) reported a patient with sensorineural deafness due to a *SERPINB6* p.E245X (c.733G>T) mutation. It's worth noting that skin diseases caused by *SERPINB6* mutations have not been reported.

## 7 Systematic evaluation of potential adverse effects associated with SERPIN modulation

Although targeting SERPINs represents a promising therapeutic strategy in skin disorders characterized by dysregulated protease activity, chronic inflammation, or impaired barrier repair, growing evidence suggests that modulation of SERPIN activity may also carry unintended consequences. These adverse effects can arise due to the pleiotropic and context-dependent functions of SERPINs across tissues and cell types (Janciauskiene et al., 2024).

For instance, overexpression of *SERPINE1*, while beneficial in limiting matrix degradation and protease-driven inflammation, has been implicated in pathological fibrosis, thrombotic complications, and delayed tissue remodeling (Ghosh and Vaughan, 2012; Flevaris and Vaughan, 2017). In animal models, PAI-1 overactivity led to excessive extracellular matrix accumulation and impaired wound healing, raising concerns about potential pro-fibrotic side effects in clinical settings. Similarly, systemic administration of *SERPINA1* is under clinical investigation for inflammatory diseases, but its broad immunosuppressive effects may increase the risk of infections or impair host defense mechanisms, especially in the skin where microbial surveillance is critical (Stockley and Turner, 2014; Greene et al., 2016).

Conversely, suppression or genetic deficiency of certain SERPINs may exacerbate tissue injury. For example, loss of *SERPINB3/4* in mice has been associated with heightened sensitivity to oxidative stress and amplified inflammatory cytokine responses (Wang et al., 2022). Given that some SERPINs play a protective role against apoptosis and oxidative damage, their inhibition may lead to collateral tissue injury, particularly under conditions of chronic inflammation or environmental stress such as UV exposure.

Importantly, SERPINs often participate in complex protease networks involving serine, cysteine, and metalloproteinases. Perturbing this delicate balance through exogenous modulation—such as gene therapy, recombinant protein administration, or small-molecule inhibitors—may disrupt homeostatic protease-antiprotease interactions, potentially leading to paradoxical effects like unrestrained proteolysis, impaired keratinocyte differentiation, or abnormal angiogenesis (Gettins and Olson, 2009). Moreover, the effects of SERPINs modulation may vary across skin compartments (e.g., epidermis vs dermis) and between physiological states (e.g., healthy vs inflamed skin), emphasizing the need for tissue-specific delivery systems and careful dose titration.

Therefore, while SERPINs constitute attractive therapeutic targets, particularly in diseases such as atopic dermatitis, psoriasis, and chronic wounds, a comprehensive risk-benefit analysis is crucial. Future research should prioritize *in vivo* safety studies using tissue-specific knockout or overexpression models, alongside rigorous pharmacokinetic and toxicological assessments of SERPINs-based therapeutics. Only through such systematic evaluation can the clinical potential of SERPINs modulation be realized while minimizing unintended harm.

## 8 Current progress and challenges in SERPIN-Targeted drug development

In recent years, *SERPINs* have emerged as promising therapeutic targets due to their central roles in regulating protease activity, inflammation, and tissue remodeling. Drug development efforts targeting SERPINs fall into several categories: recombinant SERPINs protein replacement (e.g., α1-antitrypsin for AAT deficiency), gene therapy to restore or silence *SERPINs* expression, small molecule modulators, and more recently, RNA-based therapeutics such as siRNA or antisense oligonucleotides (Cohen et al., 2019).

Recombinant SERPINA1 is among the most clinically advanced examples, approved for intravenous infusion in patients with genetic AAT deficiency-associated emphysema. However, its broader application in inflammatory skin diseases remains investigational (Yan et al., 2023). Other candidates, such as *SERPINE1* inhibitors (e.g., tiplaxtinin/PAI-039), have shown promise in preclinical models of fibrosis and thrombosis, although translation to dermatological indications has yet to be explored.

Despite encouraging progress, multiple challenges hinder the advancement of SERPIN-targeted therapies. A key concern is specificity: many *SERPINs* exhibit context-dependent and cell-type-specific functions, making broad inhibition or overexpression potentially harmful (Chen et al., 2025). Additionally, recombinant SERPINs are often large and unstable proteins, complicating delivery and limiting tissue penetration. For gene therapies, viral vectors (e.g., AAV) pose immunogenicity risks and require long-term safety validation (Kryvalap and Czyzyk, 2022). Small molecule modulators of *SERPINs* activity remain scarce, largely due to the structural complexity of *SERPINs* and the allosteric nature of their regulation. Furthermore, off-target effects and protease network compensation can undermine therapeutic efficacy.

The development of skin-targeted delivery systems (e.g., nanoparticles, microneedles, or topical formulations) and engineered *SERPINs* variants with improved stability or selectivity are actively under investigation (Wu et al., 2024; Kelly-Robinson et al., 2021). Another promising direction lies in modulating *SERPINs* expression through epigenetic or transcriptional control, which may allow finer, disease-state-specific intervention. Ultimately, translating SERPIN biology into effective dermatological therapeutics will require a multidisciplinary approach integrating molecular design, bioengineering, pharmacokinetics, and clinical trial innovation.

## 9 Conclusion

Serine protease inhibitors are critical regulators of proteolytic activity in the skin, playing essential roles in maintaining epidermal integrity, modulating inflammation, and contributing to barrier homeostasis. This review has synthesized current evidence linking mutations in specific SERPIN genes with distinct dermatological phenotypes, including ichthyosis, palmoplantar keratoderma, and psoriasis-like conditions. Beyond genetic associations, we highlighted how SERPINs influence key signaling pathways (e.g., NF-κB, MAPK, JAK/STAT) and interact with protease networks such as MMPs to regulate keratinocyte behavior and immune responses.

Although several SERPINs have emerged as potential therapeutic targets, drug development remains challenged by issues of specificity, delivery, and off-target effects. Furthermore, *in vivo* studies and transcriptomic analyses reveal cell- and context-specific expression patterns that underscore the complexity of their biological roles in skin physiology and pathology. A deeper understanding of SERPIN-regulated pathways and genotype–phenotype correlations will be essential for translating this knowledge into clinical interventions. Future work should also address the functional consequences of rare variants, clarify the pathogenic mechanisms of SERPIN dysregulation, and explore their potential as diagnostic or therapeutic biomarkers in skin diseases.
